# Poor-Law Out-Patient Departments

**Published:** 1913-07-05

**Authors:** 


### POOR-LAW OUT-PATIENT DEPARTMENTS.

It is notorious that the stay of patients in Poor-Law infirmaries is on the average much longer than those of the voluntary hospitals. The ultimate cost per patient, in spite of the simple monotonous dietary, the comparatively poor equipment of the institution, and the notorious understaffing, is therefore greater, and the ratepayers have in this way to pay for their parsimony. The ratepayers, however, owing to their want of knowledge of administration, pay the piper, others call the tune.

Many chronic cases, such as varicose ulcers of the leg, make a prolonged stay in the infirmaries. This class of case accounts for at least two large wards in each of the Metropolitan infirmaries. Some of these patients stay as long as six to ten months. If the patient is destitute, he will not be kept in the workhouse, where, owing to the small staff, administrative expenses are supposed to be low, but, because of a small chronic indolent ulcer, will be transferred, on a medical certificate, to the infirmary, where a large staff is necessary and, comparatively, the administrative expenses are naturally high. Little selection is exercised in transferring or recommending such cases to the infirmary. The cost to the ratepayers of this maladministration is enormous. Such cases may only need a daily dressing, with as much rest as possible.

To remedy this scandal an out-patient department should be attached to each Metropolitan infirmary, where such cases as chronic ulcers should be treated. The idea that the ratepayers ought to house these cases luxuriously in an expensive institution is absurd.

The abuse of the voluntary hospitals from the medical point of view is scandalous, but from the public point of view the abuse of Poor-Law institutions in this respect is even worse. As soon as the acute stage of a patient's illness is over, the custom should be, as in the voluntary hospitals, to discharge such patients for treatment in the out-patient department, provided of course that other causes do not necessitate a longer stay.

Such a scheme would not necessitate the abolition of the district medical officers. Their services must still be maintained for domiciliary visits to the destitute. But Poor-Law work at their surgeries might be discouraged in favour of the infirmary's out-patient department.

Many excellent suggestions might be made to support this scheme. Cases of ringworm in children now treated by the Metropolitan Asylums Board in institutions could be as efficiently and more economically treated in an out-patient department. Robust, healthy children are now maintained in an expensive institution at the ratepayers' expense during the treatment for patches of ringworm. These children, if treated as out-patients, could be *x*-rayed and then seen as required subsequently; the cost would be that of treatment only. Some few cases of *x*-ray dermatitis might, of course, occur which would require nursing treatment. In such cases the child could be detained for institutional treatment.

The whole apathy in this matter results mainly from the decentralised system of the Poor Law and consequent lack of administrative uniformity, but the fear of arousing the opposition of "vested interests" is a powerful deterrent to the tackling and solution of this abuse. H. B. M.

